# Multidisciplinary peer-led sexual and reproductive health education programme in France, a prospective controlled-study

**DOI:** 10.1186/s12889-022-14583-x

**Published:** 2022-12-01

**Authors:** Anaïs Nuttall, Julien Mancini, Camille Lizin, Sabrina Hamzaoui, Sophie Mariotti, Héloïse Louesdon, Sophie Tardieu, Jean-Michel Viton, Jérôme Delotte, Florence Bretelle

**Affiliations:** 1 Aix Marseille Université (AMU), AP-HM, Hôpital Conception, Hôpital de la Conception, Gynecology and obstetrics, Marseille, France; 2 Aix Marseille Univ, APHM, INSERM, IRD, SESSTIM, Hôpital Timone, Public health department (BIOSTIC), Marseille, France; 3 Université Nice Sophia Antipolis, Nice, France; 4 Réseau périnatalité Méditerranée, Marseille, France; 5 Aix Marseille Université (AMU), AP-HM, Hôpital Timone, Marseille, France

**Keywords:** Peer-led sexual education, Sexual health, Teenagers, Healthcare students

## Abstract

**Background:**

Sexual education is an international priority to promote sexual and reproductive health (SRH) and to reduce risky sexual behaviour. Experts recommend holistic and comprehensive SRH peer-led education.

In 2018, the French government launched a new public peer-led health prevention programme called the “Service Sanitaire” (SeSa), consisting of health education provided by healthcare students (peer educators) to teenagers. For the first time in France, the impact of the programme was prospectively evaluated during its first year to examine whether the programme improved the SRH knowledge of healthcare students and teenagers. Risk perception and risky sexual behaviour among these populations were also evaluated.

**Method:**

A prospective multicentre controlled study was conducted from November 2018 to May 2019. SRH knowledge was compared before and after the SeSa programme, and the evolution of this knowledge was compared, with linear regression, between healthcare students part of the SRH SeSa programme and those who were part of another programme. The same analysis of knowledge was performed with respect to teenagers who received SRH interventions as part of the SeSa compared to teenagers who did not participate in a specific SRH education programme. Risk perception and risky behaviour were studied before and after the programme among healthcare students and teenagers.

**Results:**

More than 70% of the targeted population participated in the study, with 747 healthcare students and 292 teenagers. SRH peer educators increased their knowledge score significantly more than other peer educators (a difference of 2.1 points/30 [95% CI 1.4–2.9] (p [between group] <  0.001)). Teenagers participating in the SeSa interventions also had a greater increase in their knowledge score than the other teenagers (+ 5.2/30 [95% CI 3.2–7.4] p [between group] < 0.001). There was no evidence of change in sexual risk behaviours for the healthcare student population.

**Conclusion:**

The “Service Sanitaire” programme significantly improved the sexual and reproductive health knowledge of peer-educator healthcare students and teenagers compared to a classic education programme. Longer and/or qualitative studies are needed to evaluate changes in sexual behaviour as well as positive impacts on sexuality.

**Supplementary Information:**

The online version contains supplementary material available at 10.1186/s12889-022-14583-x.

## Introduction

WHO defines Sexual and Reproductive Health (SRH) as “A state of physical, emotional, mental and social well-being in relation to sexuality; it is not merely the absence of disease, dysfunction or infirmity. SRH requires a positive and respectful approach to sexuality and sexual relationships, as well as the possibility of having pleasurable and safe sexual experiences, free of coercion, discrimination and violence” [1]. Adolescents and young adults, people who are undergoing biological and psychological change, are known to be more vulnerable and at higher risk regarding SRH [2]. In 2018, in France, 224,300 unwanted pregnancies resulted in abortions and the highest rate was among 20- to 24-year-old women [3]; moreover, detection of Sexually Transmitted infections (STIs) among the young population (15–25 years old), such as *Chlamydia trachomatis*, increased by 37% in 2 years (2016–2018) [4]. Holistic Sexual and Reproductive Health (SRH) education could be one legitimate answer for such a global public health issue [5–7]. According to the literature, school-based SRH intervention is effective in changing SRH knowledge, attitudes and skills in the young population [2, 8]. Peer-led sexual health education, education by “members of similar age or status groups” [9], is recommended [10, 11] and has proven to be efficient in increasing knowledge of sexual and reproductive health among teenagers and young adults by several studies and Cochrane reviews [12–15]. Information is more appealing and credible when provided by peers. Also, peer-led sexual education reaches two populations targeted by SRH (the educators trained by sexual education experts and the second population whom the educators teach in turn) and increases their SRH knowledge with a single programme [16]. Although peer-led SRH education has been studied, there is no study, to our knowledge, which evaluates the impact of multidisciplinary healthcare students as peer educators in SRH education.

Since 2001, French law requires that three sexual and reproductive health (SRH) education sessions be planned per year for children from 3 to 17 years old [17] despite the very poor adherence of public institutions [18]. These sessions are organized and planned by institution directors, led by school nurses, teachers or external nongovernmental organizations and their respective curricula remain greatly heterogeneous [18]. In 2018, the French government launched a new public health peer-led prevention programme called “Service Sanitaire” (SeSa) which is conducted over an academic year and mandatory for every healthcare student to obtain a degree. Until the SeSa programme, there were no mandatory nor homogenous SRH programmes implemented in public schools [18]. In 99% of French secondary schools (age 11 to 15), biology classes include SRH education as part of the human reproduction curriculum, and sessions are mostly performed by school nurses or biology teachers for teenagers at ages 13 and 14 [18]. Conversely to this approach, the SeSa programme is a peer-led SRH programme. Its aim is to teach healthcare students of all branches (medical doctors, physiotherapists, dentists, pharmacists, and midwives) priority topics (nutrition, addiction, dental hygiene and sexual health) and for these students to transfer their knowledge to secondary or primary school students [19]. Healthcare students constitute a suited peer-educator population; their shared student status and the small difference in age (5 years) with 13–15 year-old school students establishes both a relationship of trust and legitimacy between these peer educators and teenagers [9, 12]. Both populations are students and share this status, there is no authority link and both populations are in a training process [9]. Moreover, healthcare students could benefit from these interventions given their need for SRH knowledge improvement according to French and international recommendations [10, 11, 20]. In addition, healthcare students tend to have difficulties in addressing sexual subjects with patients because of a lack of knowledge [21], whereas the peer-teaching method in the medical student population has been shown as efficient in increasing SRH knowledge [22]. The short-term (one academic year) goal of the SRH SeSa programme is to improve SRH knowledge of both health care students and the public they teach to (teenagers). The long-term (several years) goal is to reduce risky sexual behaviours among these two populations and to improve SRH academic knowledge and SRH communication, advice and care among future French health professionals [20].

During the first year of the programme, we conducted a prospective study to evaluate whether the SeSa programme is efficient to increase knowledge of both healthcare students and teenager populations in SRH education with students receiving classic public health education. We also evaluated the evolution of risk perception and risky sexual behaviour among these populations over the academic year.

## Methods

### Description of the programme

The SeSa programme for healthcare students extends over one academic year and includes 20 hours of global health prevention courses, 31 hours of specific prevention (addiction, dental hygiene, nutrition and sexual education) and one to four field interventions during which healthcare students communicate their knowledge to the teenagers. Gynaecologists, SRH education experts, and governmental and nongovernmental organizations specializing in reproductive health and health education lead the 31 hours long SRH-specific programme and covered STIs (physiopathology of chlamydia, gonococcus, HPV) and HIV/AIDS information and prevention (8 hours), contraception (hormonal, non hormonal, emergency contraception, pharmaceutical aspects and technical use, 4 hours), abortion procedures (3 hours), rights and laws (4 hours), sexual and gender minority awareness (4 hours), legal aspects as well as matters of respect and consent (4 hours), sexual education communication (4 hours). Five healthcare students per group, one from each health branch (dentist, medical doctor, midwife, pharmacist and physiotherapist), lead the field interventions. The logistical aspects, duration and specific goals of these interventions are decided and organized by the schools participating in the SeSa programme. Interventions last for one to four hours for one class on one day. The participating healthcare students have to transfer their knowledge in an adapted way in order to reach the specific goals set by the relevant school.

The classic French public secondary school curriculum includes reproductive health education during the penultimate year, and school attendance is mandatory until 16 years of age. These two grades represented the most relevant groups of students to study.

### Study design

**Fig. 1:**
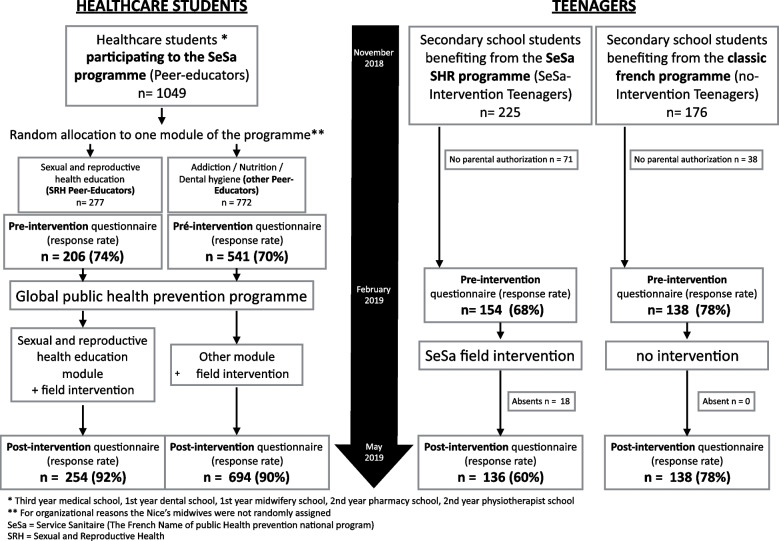
Study design

The Mediterranean Perinatology Network led this multicentric observational prospective quasi-experimental evaluation study [23] from November 2018 to June 2019 in Aix-Marseille and Nice Sophia-Antipolis Universities. Two populations were studied: the first population included healthcare students (peer-educators) participating in the SRH part of the SeSa programme (SRH peer-educators) who were compared to healthcare students participating in other modules (nutrition, addiction, and dental hygiene) (other peer-educators), and the second population included 13–15 year-old school students (teenagers) participating in the SeSa SRH programme (SeSa-intervention teenagers) who were compared to 13–15 year-old school students receiving the classic public school programme (no-intervention teenagers). Levels of knowledge, risk perception and risky behaviour were studied before and after the SeSa programme among those populations (Fig. 1). We included all students of all healthcare branches participating in the SeSa programme, as required by law, studying at the Universities of Aix-Marseille and Nice Sophia Antipolis as follows: the first year students for dental and midwifery schools, second year students for pharmacy and physiotherapy schools and third year students for medical schools. Healthcare students were randomly assigned by alphabetical order to one module of the programme, except for midwives in Nice who were assigned to the SRH module for organizational purposes. Each healthcare student participating in the SeSa programme was invited to answer a questionnaire in November 2018 before participating in any course. At the end of the academic year, in May 2019, the same questionnaire was provided again. Information on age, gender and type of studies (dentistry, medicine, midwifery, physiotherapy, and pharmacy) was collected.

For teenagers, we included voluntary secondary schools in Marseille and selected students in their penultimate and last year of secondary school (13–15 years old). Investigators invited schools included in the SeSa programme to participate in the study. After agreeing to participate, schools were assigned to the SHR group if the class participated in the SHR module and to the control group if the class participated in another module. Teenagers were invited to complete a questionnaire before the interventions in February 2019. The same questionnaire was provided three months after the end of the programme. Schools not participating in the SeSa programme received the first questionnaire in February or March 2019 and the postintervention questionnaires in May or June 2019 to respect a 3-month delay between the two questionnaires. Information on age, class, school and gender were collected.

For reasons related to participation, organization and anonymity, the data collected in this study was not paired. Whilst it had been initially planned to assign participant identification numbers this initiative was eventually abandoned given the number of participants and the lack of adherence expected if total anonymity was not ensured to both teenagers and healthcare students. Since students were part of a specific programme, we assumed that they would not change during the course of the programme. The investigators distributed the questionnaires. Neither the schoolteachers nor the university professors had access to the documents.

To participate in the study, peer educators (SRH and others) had to be part of the class of 2018 (which meant part of the SeSa programme), speak French and be over eighteen years of age. For teenagers, participation was possible if parents or legal guardians had given their consent to the study, and access to the postintervention questionnaire was not possible if the pretest questionnaire had not been completed (absent and/or no legal authorization). The distribution of participant variables is presented in Fig. 1.

### Outcome measures

The primary outcome, knowledge acquisition, was measured with 30 “true or false” questions (TFs) for healthcare students (also called peer educators) and teenagers as well as 30 additional multiple-choice questions (MCQs) for health students. The number of participants required was 1000 participants and was determined to have 80% statistical power and a bilateral significance of 5%, assuming a difference of 12% between the two groups in the postintervention score. The questions assessed knowledge of contraception use, Sexually Transmitted Infections (STIs), French SRH laws (access to abortion, free contraception, laws against homophobia, and access to pornography) and reproductive biology.

For the secondary outcome, risk perception was measured using a Likert scale [24] from 0 to 5. Participants had to decide about the level of risk of a situation, between not risky (0) and very risky [5]. Eleven situations assessed the perception of pregnancy and the transmission risk of STIs during the first intercourse, with or without a barrier protection method (condom). Information on behaviour was collected using an adapted version of the Youth Risk Behaviour Survey published by the Centers for Disease Control and Prevention’s [25]. Participants were asked to record their age at the time of their first sexual intercourse, total number of sexual partners and number of partners in the last 3 months, the contraceptive method used, if needed, and the use of alcohol or drugs, and condoms during their last sexual intercourse. The term “sexual intercourse” was not precisely defined but the French term used in the questionnaires is commonly understood as “first coit”. The participants were also asked whether they had had any “risky intercourse” (unprotected sex without knowing the STI status of their partner) during the last 2 months. The content of interventions as well as items listed in the questionnaires were checked by the UNESCO’s SERAT Tool [26] and adequacy of reporting was checked with the Tidier framework [27].

### Analysis

The quantitative data are reported as the mean ± standard deviation or as the median [minimum-maximum] and were compared using Student’s t-test or the Mann-Whitney tests. The categorical data are reported as the absolute count (percentage) and were compared using the χ^2^ test. Spearman’s rank correlation coefficient (ρ) was used to study the relationship between continuous and/or ordinal variables. The p for interaction between the timing of the questionnaire (pre- or postintervention) and the intervention (exposure or not to the SRH SeSa programme) was computed in a linear regression model to test the differential changes in quantitative outcomes among healthcare students and teenagers (p [between group]). All tests were two-sided. Differences were considered significant when the *p* value was less than 0.05. Statistical analyses were performed using IBM SPSS Statistics 20.0 (IBM Corp., Armonk, NY, USA).

### Ethical considerations

Healthcare students, 13–15 year-old school students and the legal representatives of school students gave their consent to participate in the study. The study received the approval of the French Committee for the Protection of Persons (CPP) (national number: 2018-A03066–49) and the French Data Protection Authority (national number: 2212148v0).

## Results

### Samples

Out of 1049 healthcare students (peer educators) participating in the programme, 747 questionnaires were collected pretest (71% participation rate), and 948 were collected posttest (90% participation rate) (Fig. 1). There were 277 SRH peer-educators with 206 questionnaires collected pretest (74% participation rate) and 254 responses collected posttest (92% participation rate). The responses of these peer educators were compared to those of 772 other peer-educators, with 541 questionnaires at pretest (70% participation rate) and 694 at posttest (90% participation rate). Regarding the teenagers, out of 225 participants recruited in the SeSa intervention arm, 71 participants could not provide parental authorization, 154 questionnaires were collected before the intervention (68% participation rate) and 136 after the intervention (answer rate 60%). In the no-intervention arm, 176 teenagers were recruited, 38 did not have parental authorization, and 138 questionnaires were collected pre- and posttest, respectively (78% participation rate).

As shown in Table 1, the distribution of gender, age, and age at first intercourse were similar between the SRH peer-educators and other peer-educators at baseline. The health branch representation was different between the SRH peer-educators and the other peer-educators, which was due to a higher number of midwives in the SRH peer-educator group. This result is explained by the fact that in Nice, midwives were assigned to the SRH study for organizational reasons.

**Table 1:** Sample description (at baseline)

		Sexual and reproductive health SeSa programme	Other Programme	p
***Peer-educators***		*n* = 206		*n* = 540
Age	mean (SD)	21.0 (2.1)	22.3 (2.2)	0.1
Men	n (%)	76 (36.9%)	200 (37.0%)	1.0
Women	n (%)	130 (63.1%)	340 (63.0%)	
*Studies*:				
Pharmacy	n (%)	6 (2.9%)	24 (4.5%)	< 0.001
Physiotherapy	n (%)	21 (10,2%)	76 (14.1%)	
Dentistry	n (%)	18 (8.7%)	60 (11.2%)	
Midwifery	n (%)	26 (23.6%)	15 (2.8%)	
Medicine	n (%)	135 (65.5%)	363 (67.5%)	
*City:*				
Nice	n (%)	81 (39.3%)	154 (28.5%)	0.005
Marseille	n (%)	125 (60.7%)	387 (71.5%)	
Age at first intercourse (year)	median [25–75%]	17 [16–18]	17 [16–18]	0.3
Number of lifetime sexual partner(s) (n)	median [25–75%]	3 [1–6]	2 [1–5]	< 0.001
Number of sexual partner(s) in the last 3 months (n)	median [25–75%]	1 [1–1]	1 [0–1]	0.04
***Teenagers***		*n* = 154	*n* = 138	
Age	mean (SD)	14.2 (0.5)	13.4 (0.7)	< 0.001
Boys	n (%)	79 (51.3%)	72 (52.2%)	0.9
Girls	n (%)	75(48.7%)	66 (47.8%)	
One or more lifetime episodes of intercourse	n (%)	15 (9.7%)	6 (4.3%)	0.75
One or more episodes of intercourse(s) within the last 3 months	n (%)	6 (3.9%)	3 (2.2%)	0.4

For teenagers, age was higher in the group receiving the SRH SeSa programme than in the other group (14.2 ± 0.7 y.o. vs 13.4 ± 0.5 y.o. *p* <  0.001), but the gender distribution was similar between the two groups (*p* = 0.9) (Table 1). Even if the questionnaires were not paired, there was no evidence of differences between-populations in the pre- and posttest scores for each city; neither in the gender distribution (*p* = 0.8 for Marseille and *p* = 0.7 for Nice) nor in the intervention arm (*p* = 0.9 for Marseille and *p* = 0.6 for Nice).

### Primary outcome, SeSa’s efficiency on SRH knowledge

#### Global knowledge

##### Peer-educators

There was a significant increase in the mean score of SRH peer-educators between November 2018 and May 2019. The results show an increase of 2.0 points [95% CI 1.6–2.5] out of 30 in the SRH peer-educator group vs a decrease of - 0.1 points [95% CI -0.5 – 0.3] out of 30 in the other peer-educator group for the true-false score. The difference in score between the two groups was significant, with a greater increase for SRH peer-educators than for other peer-educators (difference of 2.1 points [95% CI 1.4–2.9] (p [between group] <  0.001)). The results were similar for the multiple choice question score, with an increase of 1.6 points out of 30 [0.9–2.2] in the SRH peer-educator group vs a decrease of 1.1 points out of 30 [− 1.6 − − 0.7] in the other peer-educator group between November 2018 and May 2019. The difference in score was also significant, with a greater score increase for the SRH peer-educator group than for the other peer-educator group (difference of 2.7 [95% CI 1.8–3.6] (p [between-group] <  0.001)) (Table 2).

**Table 2:** Knowledge and risk perception results

	Students of sexual education programme				Students of other programmes					
	Before the programme mean (SD)	After the programme mean (SD)	Difference (CI 95%)	p	Before the programme mean (SD)	After the programme mean (SD)	Difference (CI 95%)	p	Diff in diff (CI 95%)	p between differences*
***Peer-educators***										
Score True-False question /30	26.5 (2.7)	28.5 (2.4)	2.0 [1.6–2.5]	< 0.001	26.3 (3.7)	26.2 (4.2)	-0.1 [-0.5–0.3]	0.74	2.1 [1.4–2.9]	< 0.001
Nice	26.9 (2.5)	29.1 (1.0)	2.2 [1.6–2.8]	< 0.001	26.2 (3.0)	26.1 (5.0)	-0.1 [-1.1–0.7]	0.76	2.3 [0.9–3.7]	0.001
Marseille	26.2 (2.8)	28.3 (2.7)	2.1 [1.5–2.7]	< 0.001	26.3 (3.9)	26.2 (3.9)	- 0.1 [-0.6–0.5]	0.83	2.2 [1.2–3.1]	< 0.001
Men	25.7 (2.9)	28.3 (2.4)	2.6 [1.8–3.4]^§^	< 0.001	25.0 (4.7)	24.9 (5.0)	-0.1 [-1.0–0.8]	0.89	2.7 [1.2–4.3]	0.001
Women	27.0 (2.4)	28.7 (2.3)	1.8 [1.2–2.3]^§^	< 0.001	27.0 (2.7)	26.9 (3.3)	-0.1 [-0.5–0.4]	0.84	1.8 [1.0–2.6]	< 0.001
Score Multiple-Choice Question/30	20.1 (3.3)	21.7 (4.0)	1.6 [0.9–2.2]	< 0.001	19.5 (3.5)	18.4 (5.0)	-1.1 [-1.6− -0.7]	< 0.001	2.7 [1.8–3.6]	< 0.001
Nice	20.4 (3.4)	23.1 (2.4)	2.7 [1.8–3-6]	< 0.001	18.2 (4.1)	16.6 (6.7)	-1.6 [2.8– -0.3]	0.01	4.3 [2.4–6.1]	< 0.001
Marseille	19.9 (3.2)	21.0 (4.4)	1.1 [0.2–2.0]	0.01	20.0 (3.1)	18.9 (4.2)	-1.1 [-1.6– -0.6]	< 0.001	2.2 [1.2–3.2]	< 0.001
Men	19.5 (2.4)	20.5 (4.3)	1.0 [-0.1−2.1]^§§^	0.06	18.0 (4.1)	17.1 (5.3)	-0.9 [-1.8–0.0]	0.05	3.2 [2.1–4.2]	< 0.001
Women	20.5 (3.6)	22.4 (3.6)	1.9 [1.1–2.7]^§§^	< 0.001	20.4 (2.7)	19.1 (4.7)	-1.3 [-1.8– -0.7]	< 0.001	1.9 [0.3–3.5]	0.02
Risk perception/5	3.4 (0.7)	3.7 (0.6)	0.3 [0.1–0.4]	< 0.001	3.3 (0.7)	3.5 (0.8)	0.2 [0.1–0.3]	< 0.001	0.1 [0.1–0.3]	0.3
Nice	3.6 (0.7)	3.8 (0.6)	0.2 [0.0–0.4]	0.05	3.3 (0.8)	3.5 (0.8)	0.2 [0.0–0.4]	< 0.001	0.0 [-0.3–0.3]	0.9
Marseille	3.3 (0.7)	3.6 (0.6)	0.3 [3.2–3.4]	< 0.001	3.3 (0.7)	3.5 (0.8)	0.2 [0.1–0.3]	0.001	0.2 [-0.4–0.3]	0.1
Men	3.2 (0.7)	3.5 (0.7)	0.3 [0.1–0.5]	0.002	3.0 (0.7)	3.3 (0.9)	0.3 [0.2–0.4]	0.001	0.1 [-0.2–0.4]	0.6
Women	3.5 (0.7)	3.8 (0.6)	0.2 [0.1–0.4]	0.002	3.5 (0.7)	3.6 (0.7)	0.1 [0.0–0.2]	0.004	0.1 [-0.1–0.3]	0.3
***Teenagers***										
Score True-False question /30	13.1 (5.9)	19.8 (6.8)	6.7 [5.2–8.2]	< 0.001	11.4 (5.6)	12.9 (7.1)	1.4 [-0.1–2.9]	0.06	5.3 [3.2–7.4]	< 0.001
Boys	12.7 (5.1)	18.2 (7.1)	5.5 [3.5–7.5]^★^	< 0.001	11.5 (5.3)	12.7 (6.9)	1.1 [-0.9–3.2]	0.3	4.4 [1.5–7.2]	0.02
Girls	13.4 (6.8)	21.3 (6.1)	7.9 [5.7–10.0]^★^	< 0.001	11.4 (6.0)	13.1 (7.4)	1.7 [-0.5–4.1]	0.14	6.1 [3.0–9.3]	< 0.001
Risk perception/5	3.1 (0.7)	3.2 (0.7)	0.1 [-0.6–0.3]	0.21	3.0 (0.9)	2.9 (0.9)	- 0.1 [-0.3–0.1]	0.30	0.2 [-0.5–0.5]	0.1
Boys	3.0 (0.7)	3.1 (0.7)	0.1 [-0.1−0.4]	0.3	2.9 (0.7)	2.8 (1.0)	-0.1 [-0.3–0.2]	0.71	0.2 [-0.2–0.5]	0.4
Girls	3.1 (0.7)	3.2 (0.8)	0.1 [-0.2−0.3]	0.5	3.2 (1.0)	3.0 (0.9)	-0.2 [-0.5–0.1]	0.27	0.3 [-0.1–0.7]	0.2

^*^ The p for interaction between the timing of questionnaire and exposure or not to SRH SeSa programme

^§^ The difference in differences between woman and men: − 0.8 [− 1.8–0.1] *p* = 0.07

^§§^ The difference in differences between women and men: + 0.9 [− 0.5–2.3] *p* = 0.2

^★^ The difference in scores’ change between boys and girls: + 2.3 [− 0.6–5.2] *p* = 0.1

##### Teenagers

Similar results were found in the teenage population, with a higher increase in the SeSa programme intervention group (+ 6.7 points out of 30 [95% CI:5.2–8.2] (*p* <  0.001) vs + 1.4/30 [95% CI: − 0.1 - 2.9] (*p* = 0.006)) (Table 2). The difference in scores was significant (*p* <  0.001).

### Specific items

#### Peer-educators

The relevant results are presented above, and the results of each question are presented in appendix 1 for true-false questions and in appendix 2 for multiple-choice questions. A higher increase in score was found for the question about the need for a pelvic examination before beginning to use hormonal contraceptives in SRH peer-educators compared to other peer-educators: 39% (*n* = 80) correct answers before the SeSa programme vs 79% (*n* = 200) after participation in the SeSa in the SRH peer-educator group, compared to 47% (*n* = 255) vs 48% (*n* = 336) in the other peer-educator group (p [between group] <  0.001) (see appendix 1). Similar results were found for questions about female virginity, legal access to abortion for minors, and emergency contraception. Scores for specific items, such as the epidemiology of abortion, technical aspects of hormonal contraception prescription and use, were low pretest, but the results showed a great increase in the SRH peer-educator group postest (mean score 0.40/1 ± 0.22 before the SeSa programme vs 0.48 ± 0.26 after p [within-group] <  0.001 for the abortion question, and a mean score of 0.39/1 ± 0.47 before SeSa vs 0.48 ± 0.25 after SeSa p [within-score] <  0.001 for the contraception question).

#### Teenagers

Girls received higher scores than boys in the post-intervention test (girls 21.3/30 – boys 18.3/30), and the mean change in score tended to be higher for girls (+ 2.3 points between boys and girls [CI 95% -0.6 – 5.2] *p* = 0.1). Knowledge increase was significantly better in the SeSa programme intervention group than in the no intervention group for questions related to the technical use of hormonal contraception (p [between group] = 0.01) and female and male pleasure (p [between group] = 0.01) (appendix 3). Higher rates of correct answers at posttest were found in the SeSa-intervention teenager group but the between-group comparison was not significant for the technical aspects of condom use (83% (*n* = 127) correct answers at pretest vs 85% (*n* = 115) at post-test in the SeSa-intervention group compared to 78% (*n* = 108) vs 72% (*n* = 99) in the no-intervention group, p [between group] = 0.24). The same results were found for knowledge of abortion laws (36% (*n* = 55) correct answers before SeSa vs 72% (*n* = 98) after SeSa in the SeSa-intervention group compared with 15% (*n* = 20) vs 34% (*n* = 47) in the no-intervention group, p [between group] = 0.29) and questions about virginity (16% (*n* = 25) correct answers before SeSa vs 43% (*n* = 59) after SeSa in the SeSa-intervention group compared to 7% (n = 10) vs 16% (*n* = 22) in the no-intervention group, p [between group] = 0.09). Despite a significant increase in the percentage of correct answers, some rates of correct answers remained low after the SeSa programme in the SeSa-intervention group for specific items, such as the role of boys in contraceptive use (36% (*n* = 55) before SeSa vs 48% (*n* = 65) after (p [within group] = 0.04)) and knowledge of the erogenous zones of boys (12% (*n* = 19) vs 41% (*n* = 56), p [within group] <  0.001). The results by question are presented in appendix 3.

### Secondary outcome, SeSa’s efficiency on risk perception and SRH behaviour

#### Peer-educators

##### Risk perception

Risk perception was significantly higher after the SeSa programme for both peer-educator groups, with a higher but not significant increase in the SRH peer-educator group (Table 2, p [between group] = 0.3]. The risk perception of each situation is presented in appendix 4. The results highlight a higher increase in the score for the risk perception of pregnancy after first intercourse (from 3.7/5 ± 1.4 to 4.21/5 ± 1.2 for SRH peer educators vs from 2.72 ± 1.4 to 3.01 ± 1.3 for other peer educators) and after condom rupture in the SRH peer-educator group (from 4.11/5 to 4.40/5 (*p* = 0.002) vs 4.03 to 4.02 (*p* = 0.89) for the other peer-educator group) (see appendix 4, p [between group] = 0.02 for both questions).

##### SRH behaviour

Ninety per cent of healthcare students reported having already had sexual intercourse, and the median age of first intercourse was 17 years of age. Among sexually-active respondents, 58.2% (*n* = 543) reported having had 3 or fewer sexual partners in their lifetime, and 63.5% (*n* = 590) only one partner during the last 3 months. When pregnancy was not desired, the most commonly reported contraceptive method at first intercourse was condoms (59%), followed by condoms combined with oral contraception (14%) and oral contraception (7%). Only 4% of respondents reported using no contraception at first intercourse, 1% reported the withdrawal technique as a contraceptive technique and 2% reported other contraceptive methods (an intrauterine device, implant, or other).

There were no significant changes in reported risky behaviours among the peer-educator population. Our study showed that 3 months after the end of the programme, there was no evidence of change in reported alcohol or drug use before last intercourse (20% before vs 13.2% after the SeSa programme *p* = 0.61), condom use during last intercourse (40.8% vs 40.4% *p* = 0.93) and last intercourse at risk (12.0% vs 14.0% *p* = 0.55) for SRH peer educators in comparison to before the programme (Table 3).

**Table 3:** Changes in risky behaviours among healthcare students (= peer educators)

	SRH peer-educators			Other peer-educators		
	Before SeSa	After SeSa	p	Before SeSa	After SeSa	p
	% (n)	% (n)		% (n)	% (n)	
**Alcohol or drug use before last intercourse**	20.0 (37/185)	13.2 (30/228)	0.61	28.5 (128/449)	23.1 (142/616)	0.43
**Condom use during last intercourse**	40.8 (75/184)	40.4 (92/228)	0.93	38.0 (171/450)	35.6 (219/615)	0.42
**« Risky** ^**a**^ **» Last intercourse**	12.0 (22/183)	14.0 (32/228)	0.55	13.1 (59/451)	14.2 (87/614)	0.61

^a^**Risky** = unprotected sex with an unknown reproductive tract infections status of a partner

### Teenagers

#### Risk perception

Risk perception tended to increase posttest in the SeSa-intervention group without reaching significance (3.07/5 ± 0.71 before the SeSa programme and 3.17/5 ± 0.74 after the SeSa programme *p* = 0.21), whereas it tended to decrease in the no-intervention group (3.03 ± 0.87 vs 2.92 ± 0.92 *p* = 0.30).

#### SRH behaviour

Given the small number of sexually active teenagers at baseline, the change in risky behaviour was not interpretable (*n* = 6 for the SeSa-intervention group and *n* = 3 for the no-intervention group).

## Discussion

### Main findings

The multidisciplinary health prevention programme “Service Sanitaire” is efficient to increase sexual and reproductive knowledge of healthcare students as peer educators and teenagers. As with other peer-led programmes, education by healthcare students is popular among teenagers given their shared student status and the more informal exchanges they can have compared to those they have with their teachers. The SeSa programme also significantly improved the sexual and reproductive risk perceptions of the healthcare students. Regarding the long-term objective of the programme, there was no evidence of impact on the risk behaviours 3 months after the acquisition of knowledge in the four groups studied (SRH peer-educators, other peer-educators, SeSa-intervention teenagers, and no-intervention teenagers). This is an expected finding giving the short duration of this study.

An unexpected finding was the reduced knowledge score posttest compared to pretest among health care students in other programmes. This result might come from a lack of interest for the subject matter and for the study being conducted at the end of the year considering the time they spent on other prevention items.

### Strengths and limitations

It should be pointed out that this study was the first to evaluate the new French SeSa programme. To our knowledge, SeSa is the only programme combining peer-led SRH education and a multidisciplinary approach at a national level. We were able to ensure the multidisciplinary approach in the evaluation, as we included and studied every branch of healthcare studies. The results were similar to those reported by other programmes [16, 28, 29] and in programme reviews [14, 30, 31]. Moreover, the Nice and Marseille scores and improvement rates were similar. Our population had the same age at the time of their first intercourse and the same type of contraceptive use as the French national population [32]. The study benefited from a high response rate, and we were able to enrol most of the 2018–2019 healthcare student classes participating in the SeSa programme. This study included and represents more than 700 peer-educators and over 300 teenagers.

The main limitation to the study is inherent to the intervention’s young teenage target population. The age at first intercourse in France is approximately 17 years old [32], and our studied teenage population was 14 years old; thus, teenagers have not yet become sexually active. In addition, it is difficult to evaluate, in a declarative mode, sexuality among teenagers. Questionnaires were distributed inside the classrooms for both populations and we reduced declarative bias by avoiding involving teachers in the process. Nevertheless, it is difficult to disregard the school’s impact on the responses. Another limitation was the unpaired data. Paired questionnaires would have ensured the correct representativeness of both groups at pretest and posttest. However, the need for total anonymity to collect sensitive data compelled us not to pair the questionnaires. As discussed and given the distribution of our population in both groups at pretest and posttest, it is more than likely that our population remains representative for both tests.

The delay between the acquisition and retention of knowledge and sexual behaviour was a limitation. A period of 4 months, as in other studies [28, 33], is too short to evaluate the behavioural impact of such a programme. This long-term objective should be evaluated with the same cohort in 5 to 6 years: teenagers would be sexually active and healthcare students would have started to exercise their profession.

### Perspectives

The SeSa programme is an interesting programme that meets the International and National Guidelines on Sexual and Reproductive Health Education [10, 18, 20]. In the Sud region (south of France) alone and all modules considered (SRH, nutrition, dental hygiene and addiction) SeSa conducted 6000 class interventions, which represents more than 150,000 students reached by this national publicly funded programme. With the same programme and at a low cost, two populations were targeted for sexual and reproductive health issues [6, 18]. Both populations will be able to use the knowledge acquired from the programme in their future lives (for teenagers) and in their future practices (for healthcare students) [34]. This study also provides ways to improve the programme. The increases in scores for items such as knowledge of abortion procedures and the epidemiology of abortion, specific aspects of hormonal contraception prescriptions and the use of these methods indicates the effectiveness of communicating information on these topics and the necessity to extend such a programme to reach a broader population. In contrast, the fact that scores on questions about the roles of boys in contraception and knowledge of the erogenous zones of boys remained low after the SeSa programme in the teenage population could lead to the improvement of this part of the programme’s curriculum. The results also show that teenage girls tend to learn better from the programme. This outcome has been pointed out in other studies [12, 16] and could suggest that more attention has to be paid to the teaching of boys to close this gap.

However, the SeSa programme does not seem to reduce risky behaviours. Although the increase in knowledge is a serious part of the process, the transformation of risky behaviour is a complex mechanism in which school is not the only component [33]. Indeed, school is currently not the only and major source of information for teenagers and young adults, as social media represents an important part of it [2]. In addition, with such a short period between knowledge acquisition and the assessment of any changes in risky behaviour, the conclusions can be misleading. This study demonstrates the effectiveness of peer-led programmes in increasing sexual and reproductive health knowledge. It could be interesting to use this type of programme combined with social media support for the young population, which has shown efficient in changing sexual health behaviour [35, 36]. An important point to make is that sexual and reproductive health is a large and global issue that is difficult to assess with evidence-based medicine. The results presented in the study evaluated a small aspect of SRH. Satisfying sexual relationships, positive sexual self-perception, partner empathy and positive preventive behaviours are many aspects composing holistic sexuality education that were taught as part of the SeSa programme and are known to be efficient and welcomed among the targeted population [37] but difficult to translate into factual indicators [33] in an institutional study (study led at school and university). These factors tend to be included in sexual health programmes and are part of the sustainable development global recommendation [6]. Further studies with a longer timeframe and/or with a qualitative approach could provide a better analysis of these factors.

## Conclusion

Our study shows that the “Service Sanitaire”, a French national health prevention programme, is efficient to increase sexual and reproductive health knowledge among healthcare students and 13–15 year-old school students. Further programmes and studies should be developed to decrease sexually risky behaviours in this population with a long-term timeframe.

## Supplementary Information

**Additional file 1.**

**Additional file 2.**

**Additional file 3.**

**Additional file 4.**

## Data Availability

Data is available upon simple request to the first author.

## Abbreviations

AIDS: Acquired Immunodeficiency Syndrome
HIV: Human Immunodeficiency Virus
HPV: Human Papilloma Virus
MCQs: Multiple-Choice Questions
SeSa: Service Sanitaire
SERAT: Sexuality Education Review and Assessment Tool
SRH: Sexual and Reproductive Health
STIs: Sexually Transmitted Infections
TFs: True or False
UNESCO: United Nations Educational, Scientific and Cultural Organization
WHO: World Health Organization
