# Supplementary material for: Multidisciplinary peer-led sexual and reproductive health education programme in France, a prospective controlled-study
Source: BMC Public Health. 2022 Dec 1;22:2239. doi: 10.1186/s12889-022-14583-x (PMC9714008; doi:10.1186/s12889-022-14583-x)
Supplement: Supplementary file 1 — Additional file 1. [file 12889_2022_14583_MOESM1_ESM.pdf]

Appendix 1 – Results of the True / False questionnaire among healthcare students (peer-educators).

	SHR peer-educators			Other peer-educators			Evolution
	Pre-intervention test	Post-intervention test	p	Pre-intervention test	Post-intervention test	p	p evolution*
	mean (SD)	mean (SD)		mean (SD)	mean (SD)		
**Score questions True-False /30**	**26.50 (2.70)**	**28.57 (2.36)**	**<0.001**	**26.25 (3.68)**	**26.17 (4,17)**	**0.74**	**< 0.001**
	**n correct answers (%)**		**p**	**n correct answer (%)**		**p**	
	**n = 206**	**n = 254**		**n = 541**	**n = 694**		
Q1 Insults and actions against homosexuals are punished by law	192 (93%)	251 (99%)	0.01	505 (93%)	654 (94%)	0.91	0.016
Q2 Pornographic films are forbidden to under 18 people	189 (92%)	244 (96%)	0.12	504 (93%)	661 (95%)	0.35	0.403
Q3 Unwanted Sexual intercourse in a married couple is not a rape	196 (95%)	247 (97%)	0.36	514 (95%)	658 (95%)	0.94	0.267
Q4 Filming or sharing sexual intercourse without protagonist conscent is forbidden even if consent is given for the intercourse	195 (95%)	244 (96%)	0.55	511 (95%)	661 (95%)	0.36	0.761
Q5 Fellation under psychological coercion is a rape	189 (92%)	247 (97%)	0.06	501 (93%)	643 (93%)	0.18	0.024
Q6 Emergency contraception is free and available without prescription for under 18 yo in pharmacy	183 (89%)	251 (99%)	<0.001	481 (89%)	610 (88%)	0.32	<0.001
Q7 Contraception also concerns boys	196 (95%)	249 (98%)	0.10	519 (96%)	658 (95%)	0.71	0.056
Q8 It is necessary to wait for the day after an intercourse with pregnancy risk to take the emergency contraception	195 (95%)	250 (98%)	0.14	484 (90%)	596 (86%)	0.15	0.010
Q9 Before pill prescription doctor has to conduct a pelvic exam	80 (39%)	200 (79%)	<0.001	255 (47%)	336 (48%)	0.03	<0.001
Q10 Minor have to have parents consent to have an abortion	140 (68%)	235 (93%)	<0.001	357 (66%)	480 (69%)	0.11	<0.001
Q11 Abortion is free and anonymous for everyone	143 (69%)	197 (78%)	0.02	359 (66%)	490 (71%)	0.13	0.364
Q12 Having contraception is possible without having sexual intercourse	201 (98%)	251 (99%)	0.75	522 (97%)	668 (96%)	1.00	0.316
Q13 The IUD (= intra-uterin device) is only for women who already gave birth	177 (86)	248 (98%)	<0.001	437 (81%)	586 (84%)	0.27	0.001
Q14 Every unprotected intercourse can lead to a pregnancy even if there is no ejaculation	164 (80%)	235 (93%)	0.00	422 (78%)	569 (82%)	0.12	0.006
Q15 It is needed to have parental authorization to get contraception	191 (93%)	245 (97%)	0.04	501 (93%)	630 (91%)	0.34	0.037
Q16 First intercourse can lead to pregnancy if it is unprotected	203 (99%)	249 (98%)	0.48	526 (97%)	658 (95%)	0.03	0.615
Q17 It is possible to know if we are affected by sexual transmitted infection for free and anonimously at any age	191 (93%)	250 (98%)	0.01	491 (91%)	641 (92%)	0.11	0.023
Q18 HIV (Human Immunodeficience Virus) / AIDS (Acquired Immunodeficiency Syndrom) is the only infection transmitted with unprotected sex	198 (96%)	248 (98%)	0.45	519 (96%)	652 (94%)	0.41	0.128
Q19 The 3 important elements to check before using a condom is : CE or NF norm, use before date, integrity of wraping	190 (92%)	248 (98%)	0.00	502 (93%)	640 (92%)	0.79	0.013
Q20 It is now possible to recover from AIDS	193 (94%)	222 (87%)	0.11	488 (90%)	591 (85%)	0.02	0.458
Q21 Oral contraception protects from STIs (Sexual-Transmitted infections)	202 (98%)	252 (99%)	0.32	519 (96%)	644 (93%)	0.14	0.095
Q22 Male condom must be use for only one intercourse. It has to be changed at each new intercourse	200 (97%)	250 (98%)	0.64	507 (94%)	656 (95%)	0.92	0.489
Q23 It is recommanded to use a condom for fellation	183 (89%)	251 (99%)	<0.001	482 (89%)	613 (88%)	0.90	<0.001
Q24 HIV can be transmitted by spittle	189 (92%)	241 (95%)	0.15	477 (88%)	592 (85%)	0.16	0.068
Q25 Clitoris is one of the female organs responsible for pleasure	204 (99%)	249 (98%)	0.48	523 (97%)	658 (95%)	0.26	0.828
Q26 Girl virginity is defined by the presence of hymen	97 (47%)	210 (83%)	<0.001	255 (47%)	365 (53%)	0.19	<0.001
Q27 Girls bleed necessarily during their first sexual intercourse	192 (93%)	251 (99%)	0.00	509 (94%)	642 (93%)	0.67	0.003
Q28 The only aim of sex is reproduction	203 (99%)	250 (98%)	0.55	523 (97%)	651 (94%)	0.10	0.486
Q29 Penis is the only erogenic zone of male body	184 (89%)	244 (96%)	0.02	487(90%)	622 (90%)	0.94	0.011
Q30 Sometime girls can be born without hymen or hymen can rend before the first intercourse	199 (97%)	249 (98%)	0.43	516 (95%)	638 (92%)	0.08	0.072

* The p for interaction between timing of questionnaire and exposition or not to SRH SeSa program
NF = French Norm / CE = European Conformity
