# Supplementary material for: Multidisciplinary peer-led sexual and reproductive health education programme in France, a prospective controlled-study
Source: BMC Public Health. 2022 Dec 1;22:2239. doi: 10.1186/s12889-022-14583-x (PMC9714008; doi:10.1186/s12889-022-14583-x)
Supplement: Supplementary file 2 — Additional file 2. [file 12889_2022_14583_MOESM2_ESM.pdf]

Appendix 2 – Results of the Multiple Choice Questionnaire in the healthcare students (peer-educators) population

	SRH peer-educators			Other peer-educators			Evolution
	Pre-intervention test	Post-intervention test	p	Pre-intervention test	Post-intervention test	p	p evolution*
	Mean (SD)			Mean (SD)			
**Total Score /30**	20.14 (3.27)	21.71 (4.00)	<0.001	19.49 (3.47)	18.35 (5.02)	<0.001	<0.001
Q1 Hormonal contraception /1	0.76 (0.20)	0.83 (0.19)	0.00	0.76 (0.20)	0.70 (0.23)	<0.001	<0.001
Q2 Sexually transmitted infections /1	0.92 (0.16)	0.91 (0.19)	0.81	0.89 (0.21)	0.83 (0.27)	<0.001	0.04
Q3 Sexually transmitted infections /1	0.72 (0.30)	0.79 (0.25)	0.01	0.70 (0.32)	0.69 (0.29)	0.06	0.02
Q4 Unprotected first intercourse /1	0.86 (0.18)	0.88 (0.19)	0.22	0.86 (0.19)	0.78 (0.27)	<0.001	<0.001
Q5 Intra-uterine device /1	0.75 (0.24)	0.84 (0.21)	0.00	0.70 (0.24)	0.68 (0.28)	0.19	<0.001
Q6 Emergency contraception /1	0.76 (0.23)	0.80 (0.20)	0.04	0.74 (0.24)	0.71 (0.27)	0.40	0.01
Q7 Human Papilloma Virus /1	0.70 (0.25)	0.74 (0.23)	0.07	0.67 (0.24)	0.64 (0.27)	0.27	0.01
Q8 Human Papilloma Virus /1	0.68 (0.25)	0.68 (0.24)	0.79	0.66 (0.24)	0.60 (0.26)	<0.001	0.02
Q9 Pregnancy risk /1	0.89 (0.17)	0.90 (0.20)	0.95	0.84 (0.21)	0.76 (0.30)	<0.001	0.003
Q10 Contraception /1	0.79 (0.18)	0.83 (0.21)	0.03	0.76 (0.21)	0.69 (0.27)	<0.001	<0.001
Q11 Sexual behaviour of adolescents /1	0.47 (0.17)	0.55 (0.20)	<0.001	0.48 (0.16)	0.49 (0.21)	0.20	0.004
Q12 Human Immunodeficiencyvirus /1	0.84 (0.16)	0.85 (0.22)	0.71	0.81 (0.20)	0.73 (0.28)	<0.001	0.001
Q13 Information source for adolescents /1	0.66 (0.19)	0.68 (0.19)	0.21	0.63 (0.20)	0.58 (0.28)	<0.001	0.002
Q14 Attitude toward fields intervention issues /1	0.74 (0.22)	0.79 (0.24)	0.04	0.74 (0.22)	0.66 (0.28)	<0.001	<0.001
Q15 Hormonal contraception /1	0.72 (0.28)	0.82 (0.24)	<0.001	0.69 (0.28)	0.66 (0.28)	0.03	<0.001
Q16 Birth under secret /1	0.65 (0.26)	0.67 (0.23)	0.33	0.63 (0.27)	0.58 (0.28)	< 0.001	0.01
Q17 Emergency contraception /1	0.52 (0.25)	0.64 (0.28)	<0.001	0.50 (0.28)	0.50 (0.29)	0.98	< 0.001
Q18 Abortion laws /1	0.60 (0.22)	0.61 (0.23)	0.70	0.55 (0.23)	0.50 (0.29)	<0.001	0.03
Q19 Abortion epidemiology /1	0.40 (0.22)	0.48 (0.26)	<0.001	0.36 (0.23)	0.40 (0.24)	0.01	0.12
Q20 Abortion procedure /1	0.59 (0.25)	0.62 (0.23)	0.25	0.59 (0.25)	0.53 (0.26)	<0.001	0.003
Q21 Abortion procedure /1	0.79 (0.25)	0.77 (0.23)	0.32	0.73 (0.26)	0.64 (0.31)	<0.001	0.02
Q22 Abortion risks /1	0.80 (0.29)	0.81 (0.29)	0.65	0.78 (0.30)	0.70 (0.35)	<0.001	0.006
Q23 Hormonal contraception /1	0.51 (0.29)	0.55 (0.28)	0.12	0.47 (0.29)	0.48 (0.28)	0.35	0.40
Q24 Hormonal contraception /1	0.46 (0.24)	0.59 (0.25)	<0.001	0.46 (0.24)	0.47 (0.26)	0.41	<0.001
Q25 Hormonal contraception /1	0.52 (0.28)	0.68 (0.25)	<0.001	0.52 (0.26)	0.51 (0.27)	0.50	<0.001
Q26 Hormonal contraception /1	0.61 (0.30)	0.71 (0.25)	<0.001	0.61 (0.29)	0.56 (0.29)	< 0.001	<0.001
Q27 Hormonal contraception /1	0.39 (0.26)	0.48 (0.25)	< 0.001	0.38 (0.24)	0.43 (0.26)	< 0.001	0.21
Q28 Menstrual cycle /1	0.73 (0.26)	0.77 (0.25)	0.10	0.70 (0.28)	0.65 (0.31)	0.06	0.006
Q29 Ovulation /1	0.56 (0.25)	0.67 (0.23)	<0.001	0.59 (0.26)	0.56 (0.27)	0.08	<0.001
Q30 Menstruation /1	0.73 (0.25)	0.79 (0.24)	0.01	0.71 (0.27)	0.65 (0.30)	<0.001	<0.001

* The p for interaction between timing of questionnaire and exposition or not to SRH SeSa program

Multiple-Choice Questionnaire

1- Regarding contraception, which items are correct :
   a. Oestro-progestativ contraception (OPC) present a higher thromboembolique risk than pregnancy
   b. Oral contraception is more efficient when taken in the morning
   c. Gaigning weight is classic with oral contraception
   d. Oral contraception has to be stopped every 6 to 12 months in order to minimise complication risk
   e. Use of condom plus pill is a safe method to avoid sexually transmitted infections (STIs) and pregnancy, it is recommanded for young women

2- Regarding STIs, which items are correct :
   a. Offering STIs screening before surgical volontary pregnancy interruption is useless
   b. Offering STIs screening before medical volontary pregnancy interruption is usefull
   c. Offering STIs screening when prescribing emergency pill is useful
   d. STIs screening is done only on urine
   e. STIs screening searchs only for Chlamydiae and Gonocoque

3- Regarding STIs, which items are correct :
   a. Infection by Neisseria Gonorrhoeae can be asymptomatic for men
   b. Infection by Neisseria Gonorrhoeae can be asymptomatic for women
   c. Infection by Chlamydia trachomatis is always asymptomatic for women
   d. Infection by Neisseria Gonorrhoeae is always asymptomatic for women
   e. Infection by Candida Albicans is a STI

4- A 16 year-old girl comes to see you after having had unprotected sex 24 hours ago with a friend. It’s their first intercourse.
   a. The girl has to take the emergency pill as soon as possible.
   b. You have to offer STI screening
   c. You have to adress the girl and her friend to have tritherapy
   d. You can suggest to start OPC after screening of contraindication
   e. It is not possible to take emergency contraception and start OPC in the same time

5- Regarding Intra-Uterine Device (IUD), which items are correct ?
   a. It is not possible for nulligest women
   b. For young women, it is better to have STI screening before placing IUD
   c. Unfertility rate are higher with IUD than with other contraceptions
   d. Uterin malformation is a contraindication of IUD
   e. IUD is efficient thanks to abortive action

6- Sylvie, 20 year-old, is on OPC. She calls you because she forgot to take a pill. She usually takes her contraception in the morning and found out the next evening. What do you advise her?
   a. She must continue her contraception
   b. If she has had intercourse within the previous 5 days, she should take emergency contraception
   c. Her contraception will no longer be effective for 15 days
   d. If she takes emergency contraception, the risk of pregnancy is zero
   e. Emergency contraception is dangerous if taken by a patient on OPC

7- Regarding Human Papilloma Virus, which items are correct?
   a. The vaccine is recommended in the vaccination schedule from 11 year old
   b. The vaccine significantly reduces the risk of cervical cancer
   c. The vaccine significantly reduces the risk of genital wart or condyloma
   d. 90% of the population will come into contact with an HPV virus
   e. Cervical cancer is the 2nd most common cancer among women worldwide

8- Regarding Human Papilloma Virus, which items are corrects
   a. Risk of cervical cancer is more common before the age of 25
   b. Tobacco is a risk factor for the persistence of the virus
   c. The vaccine is recommended for boys
   d. Pap smear is recommended at first intercourse
   e. Vaccination rate in France is 80%

9- Which items are correct?
   a. There is a risk of pregnancy when the partner withdraws before ejaculation
   b. If sex is avoided during the ovulation period, there is no risk of pregnancy
   c. You can get contraception without your parents' consent
   d. Pregnancy can happen even after having sex for the first time, even once
   e. Contraception is free for minors

10- Which items are correct?
   a. When you stop your contraception, it is effective the following month
   b. If you take contraception for too long, it can make you infertile
   c. Condom is the only effective method against Sexually Transmitted Infections (STIs)
   d. Breaking a condom during sex is a rare event (less than 1%)
   e. Contraception also concerns boys

11- Regarding adolescent sexuality, which items are correct?
   a. This sexuality is infrequent: 10 months of intercourse per year
   b. Intercourse often occurs during school vacation
   c. Intercourse is most often unprotected (over 90%)
   d. Adolescents misjudge the risk of unprotected sex
   e. The age at 1st intercourse drops steadily

12- Regarding Human immunodefficiency Virus (HIV), which items are correct?
   a. HIV can be transmitted by kissing
   b. HIV can be transmitted by unprotected oral sex
   c. Condom completely protects against transmission
   d. Risk of HIV transmission is low in adolescents and makes it possible not to use a condom in this population
   e. Risk of transmission is greater in a homosexual community

13- Regarding information sources of young population, which items are correct?
   a. Family is the main source of information
   b. One in two young people consult internet before going to the doctor
   c. Friends are a major source of information
   d. Sexual education sessions are mandatory from the age of 15
   e. General doctor is often consulted by young people

14- In the event of a declaration of a forced intercourse during a school intervention, among the following proposals which are accurate?
   a. You should inform parents of the child immediately
   b. You try to solve the problem with the young person
   c. You must, in accordance with anonymity, refer to the head of the school where you are acting
   d. You must report it to the Republic prosecutor
   e. You must suggest that the young person be received as a matter of urgency to prescribe emergency contraception and take samples

15- How is hormonal contraception taken?
   a. It can only be started on the 1st day of the period
   b. It can be started at any time during the cycle and contraception is effective from the start
   c. It can be started at any time during the cycle and contraception is effective after 7 days
   d. A delay in taking estrogen-progestogen pills is a maximum of 3 hours beyond which there is a risk of ovulation

16- Regarding childbirth under secret, what are the correct answer(s)?
   a. Any woman can request the secret of her identity within the first 3 days after birth
   b. The mother and / or the father can reconsider their decision to abandon during 1 month after the birth
   c. Access to one's origins leads to a change in civil status
   d. The woman can leave a sealed envelope with non-identifying information or with her identity for her child.
   e. After 2 months the child becomes a ward of the state and can be adopted

17- Which of the following can be offered as emergency contraception?
   a. Levonorgestrel NORLEVO 2 days after unprotected intercourse, over the counter in pharmacy
   b. Levonorgestrel NORLEVO 5 days after unprotected intercourse, over the counter in pharmacy
   c. Ulipristal ELLAONE 2 days after unprotected intercourse, over-the-counter in pharmacy
   d. Ulipristal ELLAONE 5 days after unprotected intercourse, over-the-counter in pharmacy

e. The copper intrauterine device prescribed and fitted by a doctor or midwife

18- Regarding the legal framework for voluntary termination of pregnancy (abortion) in France, what are the correct answer(s)?
   a. The law authorizing abortion in France was ratified in 1968
   b. The maximum term authorized by French law for the practice of abortion is 14 amenorrhea weeks or 12 pregnancy weeks
   c. The legal reflection period is at least 7 days after the first consultation, which can be reduced to 48 hours if there is a risk of exceeding the legal period for abortion
   d. Since 2013, abortion is beeing reimbursed at 100% for all women
   e. Unmarried minors seeking an abortion must provide parental consent

19- Regarding epidemiology, what are the correct answer(s)?
   a. The number of abortions per year has remained relatively stable for the last 15 years
   b. The number of abortions has increased over the past 15 years
   c. The proportion of repeated abortions is steadily increasing
   d. One third of women have abortion at least once in their lifetime
   e. 2/3 of abortions are requested by women using contraception

20- Regarding the abortion procedure, what are the correct answer(s)?
   a. There are two methods of abortion (medical and surgical)
   b. Surgical abortion is performed only in a health facility
   c. Medicated abortion can be done in the office by town doctors up to 7 weeks
   d. Medicated abortion can be done in the office by town doctors up to 9 weeks
   e. More than 1 in 2 abortions are now surgical abortions

21- What measures should be associated with the abortion act?
   a. Plan for later contraception
   b. Prevention of maternal-fetal blood immunization if Rh negative woman, by injection of anti-D gamma globulins within 72 hours of abortion
   c. Control consultation
   d. Psycho-affective care
   e. Routine antibiotic prophylaxis

22- What are the potential risks of a surgical abortion?
   a. Anesthetic accidents
   b. Uterine perforation
   c. Bleeding complications
   d. Tear cervix
   e. Upper genital infection: endometritis

23- Regarding the microprogestativ pill, which of the following propositions are correct(s)?
   a. The microprogestativ pill is the only pill that can be prescribed postpartum to a nursing woman
   b. No microprogestativ pill is reimbursed by social security
   c. The main contraceptive action of the microprogestativ pill is to increase the viscosity of cervical mucus
   d. The presence of a family history of venous thromboembolism contraindicates the prescription of a microprogestativ pill.
   e. The microprogestativ pill is prescribed 21 days per month

24- Regarding the estroprogesteron pill, what are the correct answer(s)?
   a. When prescribing a pill for the first time, a pelvic exam is always performed
   b. A minor patient can have the pill delivered to the pharmacy with an anonymous prescription without showing her insurrance card
   c. During a renewal consultation for an estroprogesteron pill, no clinical examination is necessary
   d. A blood test should always be done before prescribing an estroprogesteron pill
   e. The increase in migraines' frequency or intensity as well as the appearance of aura (or migraines) when taking the pill means that the pill should be stopped

25- Regarding the vaginal ring, what are the correct answer (s)?
   a. The vaginal ring should be placed and removed by a doctor or midwife
   b. The vaginal ring prevents from having sex
   c. The vaginal ring has the same contraindications as an oral estroprogestativ contraception

d. The vaginal ring is contraindicated in nulliparous patients
e. The vaginal ring may be responsible for a change in mood

26- Regarding the contraceptive patch, what are the correct answer(s)?
a. The contraceptive patch is reimbursed by social security
b. The contraceptive patch should be changed once a day
c. The contraceptive patch does not require any special biological monitoring
d. The contraceptive patch is part of estrogen-progestogen contraception
e. The contraceptive patch is often responsible of stopping the period

27- Regarding the subcutaneous hormonal implant, what are the correct answer(s)?
a. Removal of a subcutaneous hormonal implant should always be done in the operating room under general anesthesia
b. The subcutaneous hormonal implant is contraindicated in patients with a personal history of venous thromboembolic disease
c. When the subcutaneous hormonal implant is placed within the first 5 days of the hormonal cycle, it is effective straight away
d. The subcutaneous hormonal implant is contraindicated for obese women
e. The subcutaneous hormonal implant is contraindicated during breastfeeding

28- Regarding the menstrual cycle, what are the correct answer(s)?
a. The menstrual cycle usually lasts between 26 and 35 days
b. When the menstrual cycle lasts longer than 45 days, it is called spaniomenorrhea
c. The menstrual cycle can be disrupted with a significant change in weight
d. The length of the menstrual cycle does not vary throughout a woman's life
e. After the onset of the first period there may be anovulatory cycles

29- Regarding ovulation, what are the correct answer(s)?
a. Ovulation always occurs on the 14th day of the cycle in a woman whose cycles last 28 days
b. At the time of ovulation the cervical mucus becomes abundant, stringy and elastic
c. Ovulation is always accompanied by lateral pelvic pain
d. The risk of pregnancy is greater in the 1st part of the cycle than in the 2nd part of the cycle
e. Ovulation can occur either in the right or in the left ovary

30- Regarding menstruation, what are the correct answer(s)?
a. Periods lasts an average of 3 to 7 days
b. Periods correspond to the scaling of the endometrium in the absence of implantation
c. Pregnancy can happen with sex during menstruation
d. The first day of the cycle is the last day of periods
e. Periods are generally lighter and less painful on combined pill
