# Supplementary material for: Multidisciplinary peer-led sexual and reproductive health education programme in France, a prospective controlled-study
Source: BMC Public Health. 2022 Dec 1;22:2239. doi: 10.1186/s12889-022-14583-x (PMC9714008; doi:10.1186/s12889-022-14583-x)
Supplement: Supplementary file 3 — Additional file 3. [file 12889_2022_14583_MOESM3_ESM.pdf]

**Appendix 3** – **Results of the True / False questionnaire among teenagers**

| | SeSa SHR Intervention | | | No intervention | | | Evolution |
|---|---|---|---|---|---|---|---|
| | Pre-intervention test | Post-intervention test | p | Pre-intervention test | Post-intervention test | p | p evolution* |
| | mean (SD) | mean (SD) | | mean (SD) | mean (SD) | | |
| **Total Score /30** | **13.06 (5.95)** | **19.76 (6.79)** | **<0.001** | **11.44 (5.62)** | **12.87 (5.95)** | **0.07** | <0.001 |
| | n correct answer (%) n = 154 | n = 136 | p | n correct answer (%) n = 154 | n = 136 | p | |
| Q1 Insults and actions against homosexuals are punished by law | 103 (67%) | 117 (86%) | <0.001 | 111 (80%) | 117 (85%) | 0.34 | 0.06 |
| Q2 Pornographic films are forbidden to under 18 people | 127 (83%) | 123 (90%) | 0.05 | 112 (81%) | 113 (82%) | 0.88 | 0.17 |
| Q3 Unwanted Sexual intercourse in a married couple is not a rape | 80 (52%) | 99 (73%) | <0.001 | 68 (49%) | 81 (59%) | 0.12 | 0.13 |
| Q4 Filming or sharing sexual intercourse without protagonist conscent is forbidden even if consent is given for the intercourse. | 97 (63%) | 103 (76%) | 0.02 | 98 (71%) | 109 (79%) | 0.13 | 0.64 |
| Q5 Fellation under psychological coercion is a rape | 86 (56%) | 91 (67%) | 0.05 | 65 (47%) | 71 (51%) | 0.47 | 0.39 |
| Q6 Emergency contraception is free and available without prescription for under 18 yo in pharmacy | 61 (40%) | 112 (82%) | <0.001 | 33 (24%) | 69 (49%) | < 0.001 | 0.03 |
| Q7 Contraception also concerns boys | 55 (36%) | 65 (48%) | 0.04 | 50 (36%) | 53 (38%) | 0.71 | 0.24 |
| Q8 It is necessary to wait for the day after an intercourse with pregnancy risk to take the emergency contraception | 44 (29%) | 70 (52%) | <0.001 | 25 (18%) | 25 (18%) | 1.00 | 0.01 |
| Q9 Before pill prescription doctor has to conduct a pelvic exam | 25 (16%) | 64 (47%) | <0.001 | 18 (13%) | 27 (20%) | 0.14 | 0.02 |
| Q10 Minor have to have parents consent to have an abortion | 39 (25%) | 78 (57%) | <0.001 | 15 (11%) | 32 (23%) | 0.01 | 0.27 |
| Q11 Abortion is free and anonymous for everyone | 55 (36%) | 98 (72%) | <0.001 | 20 (15%) | 47 (34%) | < 0.001 | 0.29 |
| Q12 Having contraception is possible without having sexual intercourse | 58 (38%) | 82 (60%) | <0.001 | 42 (30%) | 56 (41%) | 0.08 | 0.17 |
| Q13 The IUD (= intra-uterin device) is only for women who already gave birth | 39 (25%) | 57 (42%) | 0.01 | 29 (21%) | 48 (35%) | 0.01 | 0.87 |
| Q14 Every unprotected intercourse can lead to a pregnancy even if there is no ejaculation | 37 (24%) | 72 (53%) | <0.001 | 23 (17%) | 28 (20%) | 0.44 | 0.01 |
| Q15 It is needed to have parental authorization to get contraception | 54 (35%) | 95 (70%) | <0.001 | 43 (31%) | 60 (43%) | 0.03 | 0.01 |
| Q16 First intercourse can lead to pregnancy if it is unprotected | 99 (64%) | 110 (81%) | 0.01 | 92 (67%) | 81 (59%) | 0.17 | 0.001 |
| Q17 It is possible to know if we are affected by sexual transmitted infection for free and anonimously at any age | 88 (57%) | 107 (79%) | <0.001 | 69 (50%) | 63 (46%) | 0.47 | 0.001 |
| Q18 HIV (Human Immunodeficience Virus) / AIDS (Acquired Immunodeficiency Syndrom) is the only infection transmitted with unprotected sex. | 50 (33%) | 83 (61%) | <0.001 | 52 (37%) | 52 (38%) | 0.90 | 0.001 |
| Q19 The 3 important elements to check before using a condom is : CE or NF norm, use before date, integrity of wraping. | 78 (51%) | 99 (73%) | <0.001 | 61 (44%) | 52 (38%) | 0.27 | <0.001 |
| Q20 It is now possible to recover from AIDS | 68 (44%) | 94 (69%) | <0.001 | 40 (29%) | 50 (36%) | .020 | 0.05 |
| Q21 Oral contraception protects from STIs (Sexual-Transmitted infections) | 61 (40%) | 80 (59%) | <0.001 | 49 (36%) | 55 (40%) | 0.46 | 0.09 |
| Q22 Male condom must be used for only one intercourse. It has to be changed at each new intercourse. | 127 (83%) | 115 (85%) | 0.63 | 108 (78%) | 99 (72%) | 0.21 | 0.24 |
| Q23 It is recommanded to use a condom for fellation | 57 (37%) | 89 (65%) | <0.001 | 63 (46%) | 56 (41%) | 0.40 | <0.001 |
| Q24 HIV can be transmitted by spittle | 72 (47%) | 81 (60%) | 0.03 | 43 (31%) | 53 (38%) | 0.21 | 0.56 |
| Q25 Clitoris is one of the female organs responsible for pleasure | 85 (55%) | 103 (76%) | <0.001 | 66 (48%) | 71 (51%) | 0.55 | 0.03 |
| Q26 Girl virginity is defined by the presence of hymen | 25 (16%) | 59 (43%) | <0.001 | 10 (7%) | 16 (12%) | 0.22 | 0.09 |
| Q27 Girls bleed necessarily during their first sexual intercourse | 56 (36%) | 83 (61%) | <0.001 | 32 (23%) | 41 (30%) | 0.22 | 0.07 |
| Q28 The only aim of sex is reproduction | 110 (71%) | 114 (84%) | 0.01 | 89 (65%) | 85 (62%) | 0.62 | 0.03 |
| Q29 Penis is the only erogenic zone of male body | 19 (12%) | 56 (41%) | <0.001 | 16 (12%) | 22 (16%) | 0.30 | 0.01 |
| Q30 Sometime girls can be born without hymen or hymen can rend before the first intercourse | 57 (37%) | 88 (65%) | <0.001 | 38 (28%) | 45 (33%) | 0.36 | 0.01 |

* The p for interaction between timing of questionnaire and exposition or not to SRH SeSa program
NF = French Norm / CE = European Conformity
