# Supplementary material for: Multidisciplinary peer-led sexual and reproductive health education programme in France, a prospective controlled-study
Source: BMC Public Health. 2022 Dec 1;22:2239. doi: 10.1186/s12889-022-14583-x (PMC9714008; doi:10.1186/s12889-022-14583-x)
Supplement: Supplementary file 4 — Additional file 4. [file 12889_2022_14583_MOESM4_ESM.pdf]

<u>Appendix 4.1</u> – Risk perception regarding sexual and reproductive health among peer-eduators

	SRH peer-educators				Other peer-educators				Evolution	
	Pre-intervention test	Post-intervention test	Difference (CI 95%)	p	Pre-intervention test	Post-intervention test	Difference (CI 95%)	p	Diff in Diff (CI 95%)	p
	Mean (SD)				Mean (SD)					
**Risk Evaluation /5**	3.41 (0.71)	3.78 (0.64)	0.3 [0.1 – 0.4]	<0.001	3.32 (0.75)	3.51 (0.78)	0.2 [0.1 – 0.3]	<0.001	0.1 [0.1-0.3]	0.3
R1. What is the pregnancy risk in case of unprotected sex ? /1	4.48 (0.93)	4.66 (0.71)	0.18 [0.03 – 0.33]	0.02	4.37 (0.92)	4.43 (0.95)	0.06 [-0.05 – 0.16]	0.30	0.13 [-0.07 – 0.32]	0.2
R2. What is the risk of STIs in case of unprotected sex ? /1	4.51 (0.94)	4.64 (0.80)	0.13 [-0.03 – 0.29]	0.11	4.41 (0.98)	4.48 (0.95)	0.07 [-0.04 – 0.18]	0.24	0.07 [-0.14 – 0.27]	0.5
R3. What is the risk of getting HIV in case of unprotected sexual intercourse ? /1	3.94 (1.28)	4.25 (1.13)	0.31 [0.09 – 0.54]	0.01	3.85 (1.33)	4.11 (1.21)	0.26 [0.12 – 0.41]	<0.001	0.05 [-0.22 – 0.32]	0.7
R4. What is the risk of getting HIV in case of a fellation without condom use ? /1	2.62 (1.51)	3.32 (1.51)	0.70 [0.42 – 0.98]	<0.001	2.72 (1.51)	3.09 (1.53)	0.37 [0.20 – 0.54]	<0.001	0.33 [0.01 – 0.66]	0.049
R5. What is the pregnancy risk after taking the emergency contraception 12 hours after unprotected sexual intercourse ? /1	1.36 (0.92)	1.39 (0.83)	0.03 [-0.13 – 0.19]	0.71	1.35 (0.86)	1.57 (1.08)	0.22 [0.11 – 0.33]	<0.001	- 0.19 [-0.40 – 0.02]	0.08
R6. What is the pregnancy risk after taking the emergency contraception 72 hours after unprotected sexual intercourse ? /1	2.86 (1.30)	2.91 (1.13)	0.06 [-0.17 – 0.28]	0.64	2.92 (1.14)	3.01 (1.26)	0.10 [-0.04 – 0.23]	0.17	-0.04 [-0.30 – 0.22]	0.8
R7. What is the pregnancy risk when withdrowing before ejaculation ? /1	2.89 (1.35)	3.68 (1.13)	0.79 [0.56 – 1.02]	<0.001	2.72 (1.43)	3.15 (1.36)	0.43 [0.27 – 0.58]	<0.001	0.36 [0.07 – 0.65]	0.02
R8. What is the pregnancy risk after first sexual intercourse ? /1	3.73 (1.40)	4.21 (1.16)	0.48 [0.24 – 0.72]	<0.001	3.61 (1.37)	3.72 (1.41)	0.12 [-0.04 – 0.28]	0.15	0.36 [0.06 – 0.66]	0.02
R9. What is the pregnancy risk after condom rupture ? /1	4.11 (1.04)	4.40 (0.90)	0.28 [0.10 – 0.46]	0.002	4.03 (1.10)	4.02 (1.13)	-0.01 [-0.14 – 0.12]	0.89	0.29 [0.06 – 0.52]	0.02
R10. What is the STIs risk after condom rupture ? /1	4.14 (1.06)	4.33 (0.99)	0.19 [-0.00 – 0.38]	0.05	3.99 (1.16)	4.03 (1.17)	0.05 [-0.09 – 0.18]	0.5	0.14 [-0.10 – 0.39]	0.3
R11. What is the risk of being infertile after getting an STI ? /1	2.86 (1.24)	2.68 (1.14)	-0.18 [-0.40 – 0.04]	0.11	2.53 (1.21)	2.96 (1.22)	0.43 [0.29 – 0.57]	< 0.001	- 0.61 [-0.87 - -0.34]	< 0.001

Appendix 4.2 – Risk perception regarding sexual and reproductive health among teenagers

	SeSa-Intervention				No-intervention				Evolution	
	Pre-intervention test	Post-intervention test	Difference (CI 95%)	p	Pre-intervention test	Post-intervention test	Difference (CI 95%)	p	Diff in Diff (CI 95%)	p
	Mean (SD)				Mean (SD)					
**Risk Evaluation /5**	**3.07 (0.71)**	**3.17 (0.74)**	**0.10 [-0.60 – 0.30]**	**0.21**	**3.03 (0.87)**	**2.92 (0,92)**	**- 0.11 [-0.30 – 0.10]**	**0.30**	**0.19 [-0.50 – 0.50]**	**0.1**
R1. What is the pregnancy risk in case of unprotected sex ? /1	3.85 (1.16)	4.05 (1.04)	0.20 [-0.06 – 0.46]	0.13	3.89 (1.17)	3.88 (1.14)	-0.01 [-0.28 – 0.28]	0.98	0.20 [0.18 – 0.58]	0.3
R2. What is the risk of STIs in case of unprotected sex ? /1	3.65 (1.28)	3.64 (1.27)	-0.01 [0.32 – 0.30]	0.96	3.45 (1.19)	3.33 (1.20)	- 0.13 [-0.50 – 0.25]	0.51	0.12 [-0.37 – 0.61]	0.6
R3. What is the risk of getting HIV in case of unprotected sexual intercourse ? /1	3.80 (1.20)	3.71 (1.26)	-0.09 [-0.39 – 0.21]	0.55	3.52 (1.33)	3.42 (1.27)	-0.10 [-0.47 – 0.26]	0.58	0.01 [-0.45 – 0.48]	0.9
R4. What is the risk of getting HIV in case of a fellation without condom use ? /1	2.78 (1.73)	2.89 (1.56)	0.11 [-0.31 – 0.53]	0.61	2.87 (1.57)	2.60 (1.65)	-0.27 [-0.77 – 0.24]	0.30	0.37 [-0.28 – 1.03]	0.3
R5. What is the pregnancy risk after taking the emergency contraception 12 hours after unprotected sexual intercourse ? /1	2.23 (1.53)	1.67 (1.36)	-0.56 [-0.9 – 0.19]	0.003	2.18 (1.39)	1.79 (1.28)	-0.39 [-0.79 – 0.2]	0.06	-0.17 [-0.72 – 0.38]	0.5
R6. What is the pregnancy risk after taking the emergency contraception 72 hours after unprotected sexual intercourse ? /1	3.11 (1.57)	3.25 (1.46)	0.15 [-0.24 – 0.53]	0.45	3.15 (1.41)	2.73 (1.56)	-0.42 [-0.88 – 0.04]	0.07	0.57 [-0.03 – 1.16]	0.06
R7. What is the pregnancy risk when withdrowing before ejaculation ? /1	2.05 (1.70)	2.55 (1.64)	0.50 [0.09 – 0.9]	0.02	1.55 (1.69)	1.50 (1.59)	-0.05 [-0.51 – 0.40]	0.81	0.56 [-0.56 – 1.17]	0.08
R8. What is the pregnancy risk after first sexual intercourse ? /1	2.57 (1.61)	3.31 (1.35)	0.74 [0.38 – 1.10]	< 0.01	2.57 (1.40)	2.43 (1.68)	-0.14 [-0.55 – 0.27]	0.50	0.88 [0.34 – 1.42]	0.001
R9. What is the pregnancy risk after condom rupture ? /1	3.34 (1.57)	3.52 (1.39)	0.18 [-0.18 – 0.55]	0.32	3.50 (1.35)	3.34 (1.45)	-0.16 [-0.53 – 0.21]	0.40	0.34 [-0.18 – 0.87]	0.2
R10. What is the STIs risk after condom rupture ? /1	3.11 (1.57)	3.24 (1.41)	0.13 [-0.25 – 0.50]	0.50	3.01 (1.50)	3.03 (1.53)	0.01 [-0.48 – 0.50]	0.96	0.12 [-0.49 – 0-37]	0.7
R11. What is the risk of being infertile after getting an STI ? /1	2.75 (1.49	2.67 (1.45)	-0.08 [-0.47 – 0.31]	0.68	2.67 (1.61)	2.77 (1.41)	0.11 [-044 – 0.65]	0.70	-0.19 [-0.85 – 0.46]	0.6
